# Identifying traumatic significant haemorrhage is challenging for patient with low and intermediate risk, not when bleeding is obvious

**DOI:** 10.1186/s13049-023-01096-8

**Published:** 2023-06-26

**Authors:** Vincent Darioli, François-Xavier Ageron

**Affiliations:** Department of Emergency Medicine, Lausanne University Hospital and University of Lausanne, Lausanne, 1011 Switzerland

Dear Editor,

We have read with great interest the original article of *Griggs et al.* on the predictive clinical utility of pre-hospital point of care lactate (P-LACT) for transfusion of blood products in patients with suspected traumatic haemorrhage [1]. Their work has led to the development of an algorithm aiming to identify the need for pre-hospital transfusion. We would like to address a few comments on their work.

Griggs and al consider a major haemorrhage unlikely with a P-LACT < 2.5 mmol/L. However, with a sensitivity of around 80%, almost 20% of their patients have an underestimated risk of Major Haemorrhage as assessed by their algorithm. A negative likelihood ratio of 0.37 is inappropriate to exclude a major haemorrhage as it represents a small decrease of the probability of major haemorrhage. Considering that 50% of the cohort had a P-LACT < 2.5 mmol/L, the conclusion should probably be: “Low probability of Major Haemorrhage” instead of “Major Haemorrhage unlikely”.

Given the high specificity and positive likelihood ratio of P-LACT > 6 mmol/L, it seems reasonable to conclude that pre-hospital transfusion needs to be considered. However, the proportion of patients representing this range of P-LACT is lacking to allow the reader judge the pertinence of the decision support tool. As we suspected that patients with P-LACT > 6 mmol/l are very few, the challenge for pre-hospital clinician is to identify major haemorrhage among patients with a P-LACT between 2.5 and 6 for whom P-LACT seems useless. We believe that the use of P-LACT in addition to clinical prehospital score might be useful and allows to improve clinical decision-making in the possible major haemorrhage group.

Furthermore, *Griggs et al.*. assessed the prediction of P-LACT on the in-hospital transfusion (requirement or continuation). As clinicians were not blinded from the P-LACT result, there is a high risk of circularity and false prediction. Patients with high P-LACT were probably more transfused in-hospital independently of their risk of death from bleeding. Recently, Costa et al. showed that clinical scores predicting massive transfusion were weak to predict life-threatening bleeding represented by early death and haemorrhagic death [2]. The assessment of P-LACT predicting early death within 24 h might be considered to avoid this bias.
